# Effect of the Epoxide Contents of Liquid Isoprene Rubber as a Processing Aid on the Properties of Silica-Filled Natural Rubber Compounds

**DOI:** 10.3390/polym13183026

**Published:** 2021-09-07

**Authors:** Gyeongchan Ryu, Donghyuk Kim, Sanghoon Song, Kiwon Hwang, Wonho Kim

**Affiliations:** 1 School of Chemical Engineering, Pusan National University, Busan 46241, Korea; 60chan95@gmail.com (G.R.); ehdgurzxc@gmail.com (D.K.); thdtkd1111@gmail.com (S.S.); 2 Hankook Tire & Technology Co., Ltd., R&D Center, Daejeon 34127, Korea; kiwon8348@gmail.com

**Keywords:** natural rubber, processing aid, epoxidized liquid isoprene rubber, silica-filled compound, filler–rubber interaction

## Abstract

In this study, we examined the feasibility of using epoxidized liquid isoprene rubber (E-LqIR) as a processing aid for truck and bus radial (TBR) tire treads and investigated the effects of the epoxide content on the wear resistance, fuel efficiency, and resistance to extraction of the E-LqIRs. The results confirmed that, compared to the treated distillate aromatic extract (TDAE) oil, the E-LqIRs could enhance the filler–rubber interactions and reduce the oil migration. However, the consumption of sulfur by the E-LqIRs resulted in a lower crosslink density compared to that of the TDAE oil, and the higher epoxide content decreased the wear resistance and fuel efficiency because of the increased glass-transition temperature (*T*_g_). In contrast, the E-LqIR with a low epoxide content of 6 mol% had no significant effect on the *T*_g_ of the final compound and resulted in superior wear resistance and fuel efficiency, compared to those shown by TDAE oil, because of the higher filler–rubber interactions.

## 1. Introduction

Significant efforts have been made to increase the fuel efficiency and wear resistance of truck and bus radial (TBR) tires owing to the recent environmental regulations and emergence of electric vehicles [1]. The tire tread is the only part of the vehicle that is in contact with the ground. Therefore, the rolling resistance of the tire tread greatly affects the fuel efficiency of the vehicle, and the wear resistance of the tire tread is important for the long-term use of the tire. Moreover, vehicles such as trucks and buses typically carry heavy loads and are used for long-distance transportation; consequently, it is necessary to increase the fuel efficiency and wear resistance of TBR tires. One such effort involves the replacement of the carbon black that is used in TBR tire treads with silica and silane coupling agent (SCA) [2,3,4]. The TBR tire treads generally use natural rubber (NR) as the base rubber; however, the silica–SCA–NR interactions are weak because of the interference by the proteins and lipids present in NR [5,6]. These weak filler–rubber interactions decrease the wear resistance of the TBR tires and, as a result, the use of silica in TBR tire treads is limited compared to that of carbon black.

In addition, with the recent advances in autonomous driving, it is expected that automated cargo trucks can be operated for 24 h a day. As a result, another issue with TBR tire is that processing aids such as TDAE oil migrate to the tire surface over time, resulting in deterioration of the physical properties of the vulcanizates [7,8]. Liquid rubber has received considerable interest as a new processing aid to solve the oil migration problem. Its applicability as a processing aid in passenger car radial tires has been actively studied in recent times, confirming that the use of liquid rubber as a processing aid can improve the processability and alleviate the oil migration problems [9,10,11]. However, studies on the application of liquid rubber in TBR tire treads have not been reported to date; moreover, no quantitative analysis has been conducted on the effect of liquid rubber on the vulcanizate structure of these compounds.

In addition, it has been reported that the low filler–rubber interactions of the silica-filled NR compounds can be increased through chemical interactions between the hydroxyl groups of silica and epoxide group of rubber by introducing epoxide groups in the NR [12]. Such increased filler–rubber interaction can be improved wear resistance.

In this study, E-LqIRs with different epoxide contents were prepared. E-LqIRs were used as a processing aid, and the properties of the compounds according to the epoxide contents were compared. E-LqIR can improve not only wear resistance but also the oil migration problem. Therefore, E-LqIR can be expected to have excellent properties for the TBR tire tread. However, no study has been conducted on how liquid rubber with high functionality such as E-LqIR acts on the vulcanizate structure of compounds. Therefore, we tried to quantitatively analyze the effect of E-LqIRs on the vulcanizate according to the epoxide contents through vulcanizate structure analysis.

## 2. Materials and Methods

### 2.1. Materials

#### 2.1.1. Polymerization

All the materials were purged with nitrogen. Cyclohexane (99%, Samchun Chemical Co., Seoul, Korea) was used as the organic solvent, and *n*-butyllithium (2.0 mol/L in cyclohexane, Sigma–Aldrich Corp., Seoul, Korea) was the anionic initiator. Isoprene (99%, Samchun Chemical Co., Seoul, Korea) was used as a monomer, and tetrahydrofuran (THF; 99%, Duksan General Chemical Co., Seoul, Korea) was used as a polar modifier to increase the reaction rate. In addition, *n*-octyl alcohol (99%, Yakuri Pure Chemicals Co. Ltd., Kyoto, Japan) was used as the termination agent.

#### 2.1.2. Epoxidation

LqIR was converted to the epoxidized form, i.e., E-LqIR using hydrogen peroxide (30 wt% in H_2_O, Sigma–Aldrich Corp., Seoul, Korea) and formic acid (98%, Sigma–Aldrich Corp., Seoul, Korea).

#### 2.1.3. Compounding

NR (Standard Vietnamese Rubber SVR-10, dirt content = 0.1 wt%; Astlett Rubber Inc., Oakville, ON, Canada) was used as the base rubber. Silica (Ultrasil 7000 GR, Evonik Industries AG, Essen, Germany), with a Brunauer–Emmett–Teller surface area of 160–175 m^2^/g, was used as the filler. Bis [3-(triethoxysilyl)propyl]-tetrasulfide (TESPT, Si-69; Evonik Korea Ltd., Seoul, Korea) was used as the silane coupling agent. ZnO and stearic acid (Sigma–Aldrich Corp., Seoul, Korea) were used as activators, and *N*-(1,3-dimethyl butyl)-*N*-phenyl-*p*-phenylenediamine (6PPD, Kumho Petrochemical Co., Daejeon, Korea) was used as the antioxidant. Sulfur (Daejung Chemicals and Metals Co., Siheung, Korea) was used as the crosslinking agent. *N*-cyclohexylbenzothiazyl-2-sulfenamide (CBS; 98%, Tokyo Chemical Industry Co., Ltd., Tokyo, Japan) and 1,3-diphenylguanidine (DPG; 98%, Tokyo Chemical Industry Co., Ltd., Tokyo, Japan) were used as the curing accelerators. *N*-(cyclohexylthio)phthalimide (PVI, Shandong Yanggu Huatai Chemical Co., Ltd., Liaocheng, China) was used as the prevulcanization inhibitor.

### 2.2. Measurements

#### 2.2.1. Gel Permeation Chromatography

The molecular weight and its distribution were measured using a gel permeation chromatography (GPC) system (Shimadzu, Kyoto, Japan). The GPC system consisted of a solvent delivery unit, refractive index detector, and three types of Styragel columns: HT 6E (10 µm, 7.8 mm × 300 mm), HMW 7 column (15–20 μm, 7.8 mm × 300 mm), and HMW 6E column (15–20 μm 7.8 mm × 300 mm). The measured molecular weight was corrected using a polyisoprene standard sample (Polyisoprene Standard Kit, Waters Corp., Milford, MA, USA).

#### 2.2.2. H Nuclear Magnetic Resonance Spectroscopy (^1^H NMR)

The epoxide content of the E-LqIR samples was determined by ^1^H NMR (Varian, Unity Plus 300, Garden State Scientific, Morristown, NJ, USA). Solutions of E-LqIRs (15 mg/mL) in deuterated chloroform (CDCl_3_, Cambridge Isotope Laboratories, Inc., Andover, MA, USA), prepared in 5 mm NMR tubes, were used for measuring the spectra.

#### 2.2.3. Differential Scanning Calorimetry

The glass-transition temperatures (*T*_g_) of the E-LqIRs were measured by differential scanning calorimetry (DSC; DSC-Q10, TA Instruments, New Castle, DE, USA). The E-LqIR samples (3–6 mg) were analyzed from −80 to 100 °C at a heating rate of 10 °C/min.

#### 2.2.4. Payne Effect

The degree of filler–filler interactions of the compounds was determined by following the standard procedure described in ASTM D8059, using a rubber processing analyzer (RPA2000, Alpha Technologies, Hudson, OH, USA). The storage modulus (*G′*) of the compounds after the first stage of mixing was measured at 60 °C within a strain range of 0.01–40.04%. The silica agglomerates did not disintegrate under a low strain; thus, the storage modulus was high in the low strain region and decreased when a higher strain was applied. This is called the Payne effect, which can be quantified by the change in the storage modulus (Δ*G′*), and represents the degree of filler–filler interaction. In this study, Δ*G′* was calculated by subtracting the value at a strain of 40.04% from that at 0.28% and was used as an indicator of the degree of filler dispersion within the rubber compounds.

#### 2.2.5. Mooney Viscosity

The processability of the rubber compound was evaluated using the standard procedure described in ASTM D164. The sample was preheated to 100 °C for 1 min. Next, a Mooney viscometer (VluChem Ind Co., Seoul, Korea) was used to measure the torque produced by a rotor rotating at 2 rpm for 4 min within a space filled with unvulcanized rubber.

#### 2.2.6. Bound Rubber Content

After the first mixing stage, a sample of each compound (0.2 ± 0.01 g) was placed on a filter paper and immersed in toluene (100 mL) for 6 days at 25 °C to extract the unbound rubber. Next, the toluene contained in the extracted unbound rubber was cleaned with acetone and dried. The bound rubber content was computed based on the sample weights before and after the experiment using Equation (1) as follows:

(1) $$Bound\;rubber\;content\;\left(\%\right)=\frac{w_{fg}-w_{t} [\frac{m_{f}}{m_{f}+m_{r}}]}{w_{t} [\frac{m_{r}}{m_{f}+m_{r}}]}\times 100$$

Here, *w_fg_* is the combined weight of the filler and gel, *w_t_* is the weight of the specimen, *m_f_* is the weight fraction of the filler in the compounds, and *m_r_* is the weight fraction of the polymer in the compounds.

#### 2.2.7. Curing Characteristics

The curing characteristics of the compounds were evaluated using the minimum torque (*T*_min_), maximum torque (*T*_max_), scorch time (*t*_10_), and optimal curing time (*t*_90_). These characteristics were measured using a moving die rheometer (MDR; Myung Ji Co., Seoul, Korea) at a vibration angle of ±1° and temperature of 150 °C, over 30 min.

#### 2.2.8. Crosslink Density and Vulcanizate Structure Analysis

The vulcanized specimens (10 mm × 10 mm × 2 mm) were sequentially immersed in THF (99%, Samchun Chemical Co., Seoul, Korea) and *n*-Hexane (95%, Samchun Chemical Co., Seoul, Korea) at 25 °C for 1 day to remove any organic additives in the specimens. The weights of these treated specimens were then recorded. Next, the specimens were immersed in toluene at room temperature for 1 day, and the resulting swollen specimens were weighed. The total crosslink density was calculated using the Flory–Rehner equation (Equation (2)) expressed as

(2) $$\nu =\frac{1}{2M_{c}}=-\frac{\ln\left(1-V_{r}\right)+V_{r}+\chi V_{r}^{2}}{2\rho_{r}V_{s}\left(V_{r}^{\frac{1}{3}}-0.5V_{r}\right)}$$

Here, *ν* is the crosslink density (mol/g), *M_c_* is the average molecular weight between the crosslink points (g/mol), *V_r_* is the volume fraction of rubber in the swollen gel at equilibrium, vs. is the molar volume of the solvent (cm^3^/mol), *ρ_r_* is the density of the rubber vulcanizates (g/cm^3^), and *χ* is the polymer–solvent interaction parameter. Furthermore, the chemical crosslink density of the unfilled compounds was calculated using the Flory–Rehner (Equation (2)) and Kraus equations (Equation (3)).

(3) $$\frac{V_{r0}}{V_{r}}=1-m\left(\frac{\varphi }{1-\varphi }\right)$$

Here, *V_r0_* is the volume fraction of unfilled rubber in the swollen gel at equilibrium, *V_r_* is the volume fraction of rubber in the swollen gel at equilibrium, and *φ* is the volume fraction of the filler_._ Subsequently, the degree of filler–rubber interaction was calculated as the difference between the total crosslink density (chemical crosslink density + filler–rubber interaction) and the chemical crosslink density.

#### 2.2.9. Mechanical Properties

Dumbbell-shaped vulcanizate specimens (length = 100 mm; width = 25 mm) were tested at a speed of 500 mm/min using a universal testing machine (UTM, KSU-05M-C, KSU Co., Ansan, Korea) to evaluate their mechanical properties, including the tensile strength, modulus, and elongation at break. The sample testing was performed according to the standard procedure described in ATSM D 412.

#### 2.2.10. Abrasion Resistance

The abrasion resistance was measured using an abrasion tester (DIN: Deutsche Industrie Normen, KSU Co., Ansan, Korea) according to the standard procedure described in DIN 53516. In this process, an abrasive sheet was rotated on the surface of cylindrical specimens (diameter = 16 mm; length = 8 mm) at 40 ± 1 rpm under a load of 5 N, and the subsequent mass loss was measured.

#### 2.2.11. Viscoelastic Properties

The viscoelastic properties of the compounds were evaluated by measuring the storage modulus (*E*), loss modulus (*E*″), and tan δ at 0.2% strain and 10 Hz using a dynamic mechanical analyzer (DMA Q800, TA Instrument, New Castle, DE, USA), between −80 and 100 °C.

### 2.3. Synthesis of Epoxidized Liquid Isoprene Rubbers

**Scheme 1:**
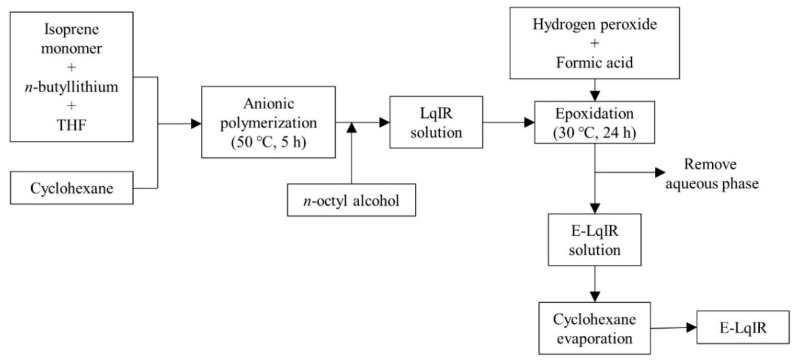
Schematic flow chart of the E-LqIR synthesis process.

**Scheme 2:**
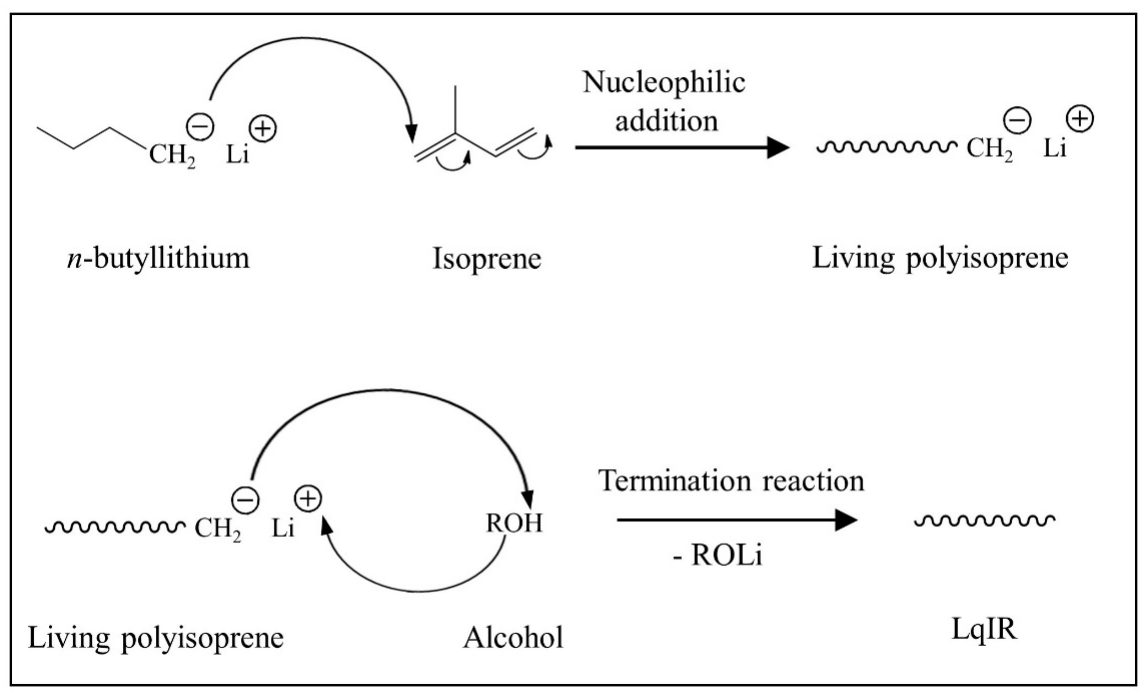
Polymerization of LqIR.

**Scheme 3:**
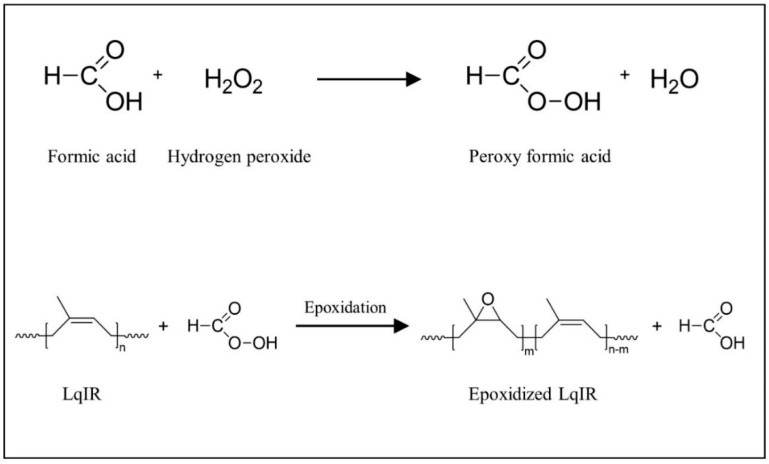
Epoxidation of LqIR.

The whole synthesis process is shown in Scheme 1. At first, LqIR was synthesized by anionic polymerization in a nitrogen-purged reactor at 50 °C. Cyclohexane and *n*-butyllithium were used as solvent and initiator, respectively. THF was added at a molar ratio of 0.25 relative to the initiator to accelerate the reaction [13]. Subsequently, isoprene was introduced into the reactor under nitrogen atmosphere. The polymerization of LqIR lasted 5 h, and then the reaction was terminated by adding *n*-octyl alcohol (1.2 M; in excess with respect to the initiator) (Scheme 2). After terminating the reaction, the LqIR solution was removed from the reactor and placed in a three-necked round-bottom flask along with aqueous hydrogen peroxide and formic acid. The heterogeneous solution containing the cyclohexane and aqueous phases was stirred at a speed of 1000 rpm using a high-speed stirrer at 30 °C and allowed to react at the suspension interface for 24 h (Scheme 3) [14]. The E-LqIRs were prepared with various epoxide contents by adjusting the ratios of hydrogen peroxide and formic acid. After the epoxidation reaction, the aqueous phase containing hydrogen peroxide and formic acid was removed to prevent residual reaction, and the E-LqIRs were obtained by evaporating cyclohexane from the E-LqIR solution using a vacuum evaporator. The macrostructures and microstructures of the E-LqIRs were analyzed by GPC and ^1^H NMR spectroscopy, and *T*_g_ was measured using DSC.

### 2.4. Preparation of Rubber Compounds and Vulcanizates

The rubber compounds were synthesized using an internal mixer (300 cc, Mirae Scientific Instruments Inc., Gwangju, Korea) and the formulations presented in Table 1. A fill factor of 80% of the mixer volume was used. The input unit was parts per hundred rubber (phr), and the compounds were added in proportions relative to the amount of rubber.

The mixing procedure is outlined in Table 2. For the first and second stages, the initial temperatures were 100 and 50 °C, respectively, and the dump temperature ranges were 150–155 and 80–90 °C, respectively. After mixing was completed in each stage, the compounds were transformed into sheets using a two-roll mill. Finally, the vulcanizates were prepared by pressing the compounds in a hydraulic press at 150 °C, for the optimal curing time (*t*_90_).

## 3. Results and Discussion

### 3.1. Synthesis of Epoxidized LqIR

**Figure 1:**
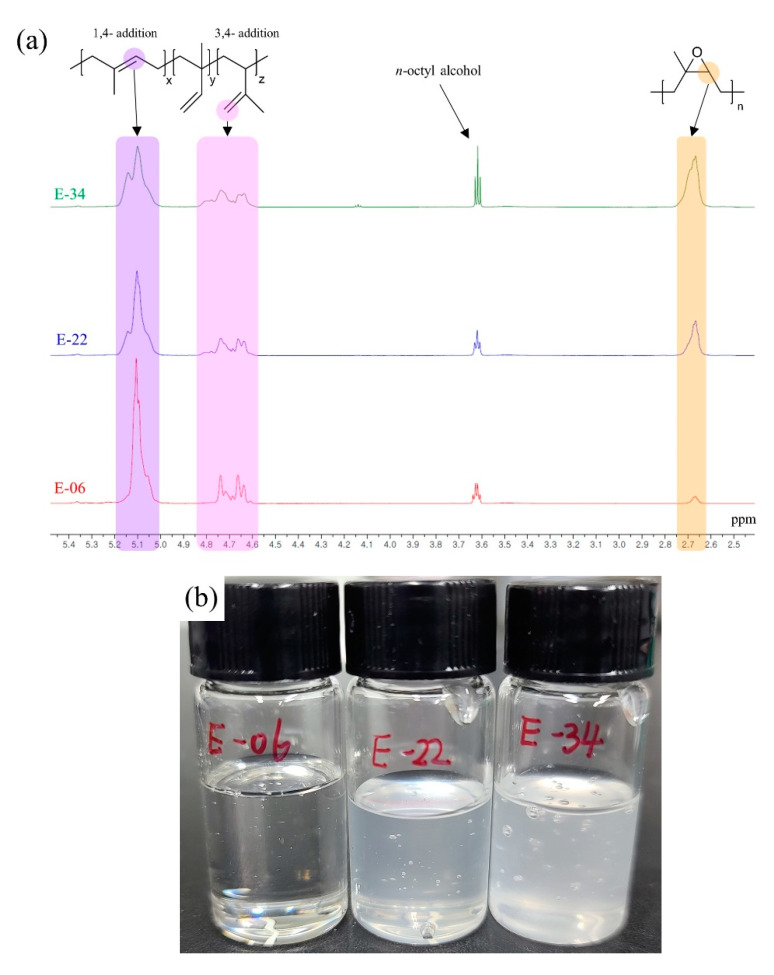
Synthesized E-LqIRs: (**a**) ^1^H NMR spectra, and (**b**) photographic image.

To prepare the E-LqIRs as processing aids, a low-molecular-weight LqIR (*M*_n_: 3639 g/mol) having excellent flow properties was polymerized [15]. After that, the LqIR was used for the preparation of E-LqIRs. E-LqIRs with different epoxide contents was prepared by varying the contents of formic acid and hydrogen peroxide. Figure 1 and Table 3 show the GPC, ^1^H NMR, and DSC results of the E-LqIRs. The GPC results indicate that as the epoxide contents increased, the molecular weight increased due to interactions such as hydrogen bonding and self-crosslink between E-LqIRs [16,17]. In the ^1^H NMR spectra, signals were seen at 4.6–4.8, 4.8–5.0, and 5.0–5.2 ppm owing to the olefinic methine protons of the 3,4-, 1,2-, 1,4-addition units of polyisoprene, respectively. The signals originating from epoxy methane protons were observed at 2.7 ppm [18,19]. Additionally, signals arising from the *n*-octyl alcohol, added as a reaction terminator for LqIR, were observed at 3.6 ppm. The epoxide contents of the E-LqIRs were calculated using the areas of the peaks at 4.6–4.8, 5.0–5.2, and 2.7 ppm (Equation (4)) and found to be 6.0, 22.1, and 34.4 mol%, respectively. The results showed that the epoxide content increased with decreasing 1,4-addition content.

(4) $$Epoxide\;contents\;\left(mol\;\%\right)=\frac{A_{2.7}}{A_{2.7}+A_{5.0\sim 5.2}+A_{4.6\sim 4.8}/2}\times 100$$

where *A* represents the area of each peak corresponding to different concentrations.

The *T*_g_ values of the E-LqIRs were measured using DSC and were found to increase with increasing epoxide content as a result of the interactions between the epoxide groups. These interactions limited the chain mobility [20,21]; consequently, the *T*_g_ values of the E-LqIRs increased by 0.78–0.88 °C for every 1 mol% increase in the epoxide content. The resulting *T*_g_ values were −72.46, −60.28, and −47.44 °C.

The functionality—the number of epoxide groups for every E-LqIR chain—was calculated using the GPC and NMR results. The average unit number of chains was calculated (Equation (5)) using the molecular weights of the isoprene unit (68.12 g/mol) and epoxidized isoprene unit (84.12 g/mol) as well as *M*_n_. The average number of epoxide groups per chain was calculated (Equation (6)) using the epoxide content obtained from Equation (4).

(5) $$Chain\;unit\;number=\frac{M_{n}}{\left(68.12\times \left(1-\frac{epoxide\;contents}{100}\right)+84.12\times \frac{epoxide\;contents}{100}\right)}$$

(6) $$unctionality\;\left(Epoxide/Chain\right)=Chain\;unit\;number\times \frac{epoxide\;contents}{100}$$

**Figure 2:**
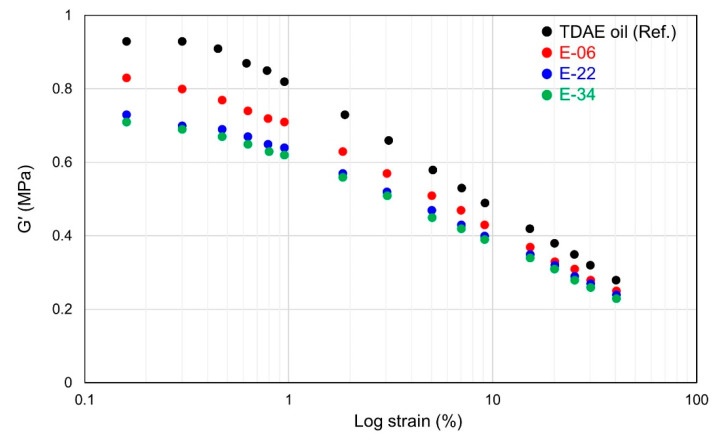
Strain dependences of the shear storage modulus (*G*′) for the compounds.

The Payne effect analysis, shown in Figure 2 and Table 4, revealed the existence of filler–filler interactions in the uncured compounds [22,23]. In general, the storage modulus (*G*′) reduces with increasing strain amplitude as a result of the breakdown of the filler–filler network; however, a higher Δ*G*′ value represents a stronger filler–filler interaction. Therefore, the degree of silica dispersion was determined based on the Δ*G*′ values because a low Δ*G*′ implies better silica dispersion within a compound. The results showed that the Δ*G*′ value of the E-LqIR compounds were smaller than that of the TDAE oil compound because the epoxide group in the E-LqIR interacts with silanol group on the silica surface in the E-LqIR compounds. Therefore, it was confirmed that the dispersion of silica was improved by increasing the epoxide content of the E-LqIRs.

### 3.2. Curing Characteristics and Mooney Viscosity

**Figure 3:**
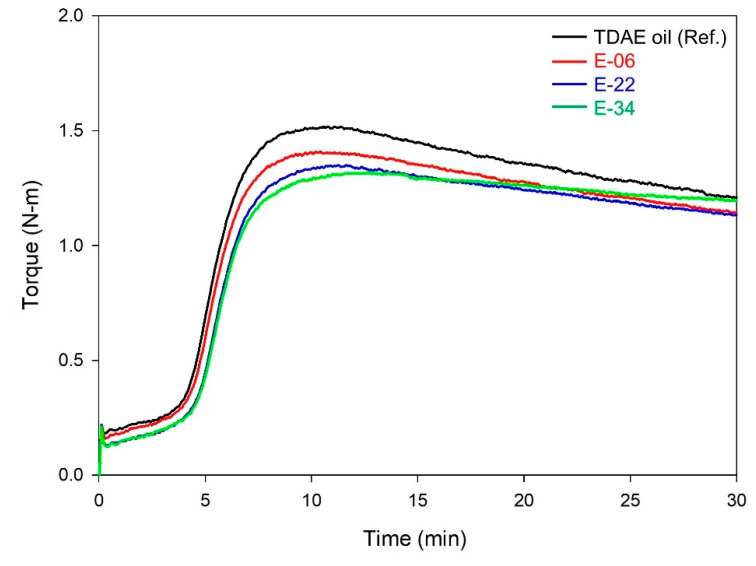
Curing characteristics determined by MDR at 150 °C.

Figure 3 and Table 5 show the results of Mooney viscosity, bound rubber, and curing characteristic measurements. Increasing the epoxide content led to higher silica dispersion and decreased the Mooney viscosity and *T*_min_ values. Furthermore, decreasing the occluded rubber content reduced the bound rubber contents [24]. The curing characteristic measurements revealed that the Δ*T* value of the E-LqIRs was smaller than that of the TDAE oil compound because of the consumption of sulfur by the double bonds of E-LqIRs. On the other hand, as the number of double bonds of E-LqIRs decreased with increasing epoxide content, the amount of E-LqIR that could have reacted with the NR via sulfur also decreased, resulting in a smaller Δ*T* value.

### 3.3. Crosslink Density and Vulcanizate Structure Analysis

**Figure 4:**
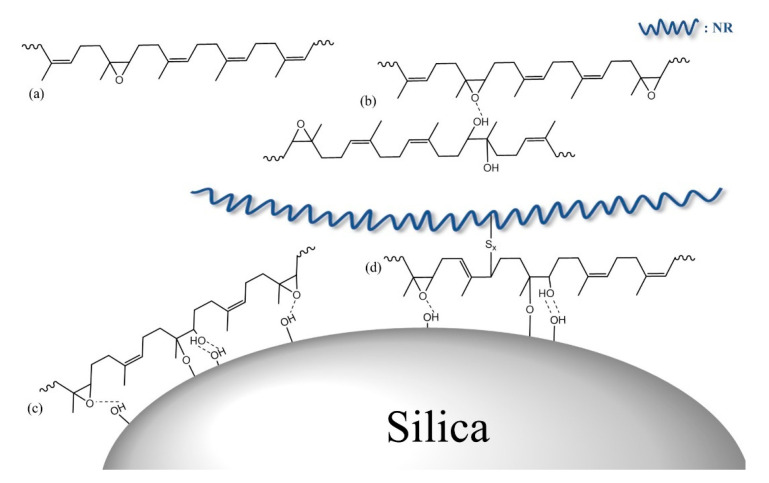
Proposed interactions of silica-filled NR vulcanizates with E-LqIRs: (**a**) E-LqIRs without interaction with silica and rubber, (**b**) hydrogen bonding of E-LqIRs via ring-opened epoxide groups, (**c**) E-LqIR as a silica covering agent, and (**d**) E-LqIR as a coupling agent owing to sulfur crosslinking.

Figure 4 illustrates the proposed interactions of the E-LqIRs with the silica-filled NR compound vulcanizates. The E-LqIRs that do not interact with silica (Figure 4a) can act as lubricants. These E-LqIRs can also be extracted during the pretreatment stage of the swelling tests owing to the absence of interactions. In addition, when ring-opening occurs, an interaction between E-LqIRs is formed (Figure 4b), which increases the molecular weight of E-LqIRs. The E-LqIRs that form hydrogen bonds and direct silica–epoxy bonds with the hydroxyl group of silica (Figure 4c) can act as silica-covering agents. These E-LqIRs cannot be extracted during the pretreatment stage of the swelling tests [25]. In addition, some E-LqIRs that interact with silica can become coupled with the NR through crosslinking with sulfur and can act as coupling agents (Figure 4d).

**Figure 5:**
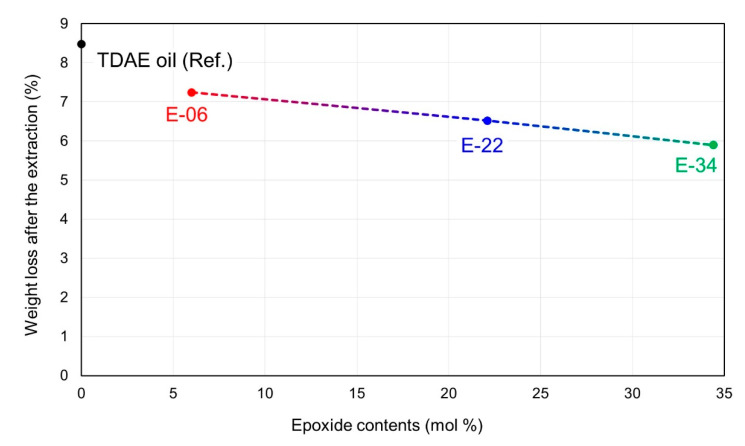
Weight loss after extraction with respect to the epoxide contents of the E-LqIRs.

During the pretreatment stage of the swelling tests, the organic matter present in the vulcanizates was extracted using two different organic solvents: THF and *n*-hexane. Figure 5 and Table 6 show the amounts of extracted organic matter. The proportion of 10 phr of TDAE oils in the vulcanizate specimen was 5.44 wt%. The TDAE compound showed the highest organic matter extraction amount as 8.48 wt% (10 phr of TDAE oil; 5.44 wt% + some additives such as stearic acid, 6PPD, TMQ, etc.; 3.04 wt%). This is because the oil can easily be extracted by organic solvents, as it does not form a chemical bond with other materials in the compound. However, the E-LqIRs were more resistant to extraction because of the interactions of the epoxide groups with silica. Assuming that the TDAE oil was completely extracted, and the amounts of additives extracted is the same, the amounts of E-LqIRs extracted decreased with increasing epoxide content (i.e., 66.6%, 53.2%, and 41.8% for E-06, E-22, and E-34, respectively). This result confirms that increasing the epoxide content of E-LqIRs will enable their use as processing aids in tires by reducing the oil migration problem in tires [8].

**Figure 6:**
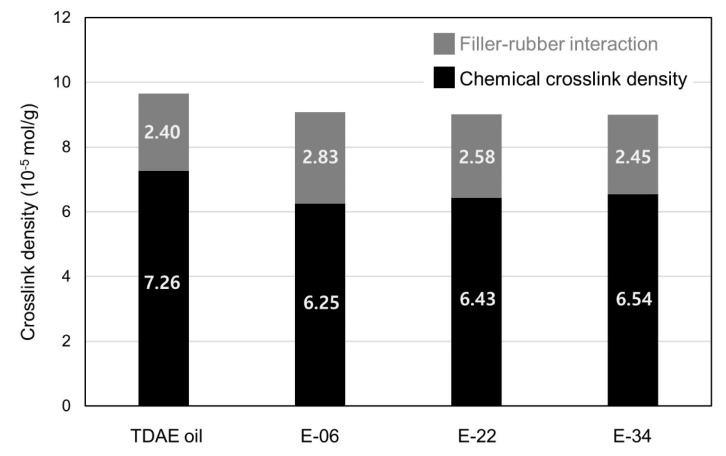
Crosslink densities of the vulcanizates.

To determine the effect of the epoxide content of E-LqIRs on the vulcanizate structures of the rubber compounds having various filler contents, the total crosslink density was calculated as the sum of the filler–rubber interactions and chemical crosslink density via vulcanizate structure analysis [25,26,27,28,29,30,31,32,33]. Figure 6 and Table 6 present the results of the analysis. The chemical crosslink density obtained by adding E-LqIRs was lower than that obtained by adding TDAE oil because of the additional consumption of sulfur by the double bonds of the E-LqIRs. On the other hand, more filler–rubber interactions occurred in the E-LqIR compounds than in the TDAE oil compound because of sulfur, which acted as a coupling agent with the E-LqIRs. A higher epoxide content increased the amount of E-LqIRs covering the silica surface, thereby reducing the absorption of the cure accelerator on the silica surface [25]. Furthermore, additional sulfur consumption decreased because of the decrease in the number of double bonds in the E-LqIRs. As a result, the chemical crosslinking density increased. On the other hand, the higher epoxide content reduced the filler–rubber interactions because the number of double bonds available for crosslinking with sulfur decreased.

### 3.4. Mechanical Properties and DIN Abrasion Loss

**Figure 7:**
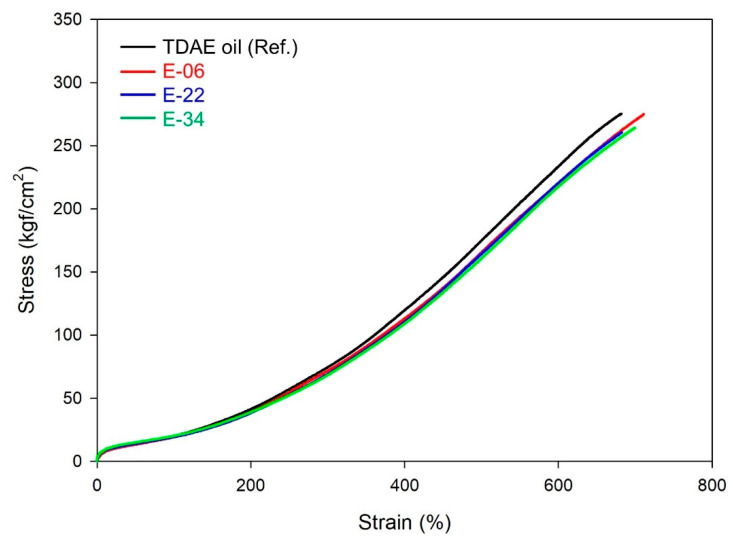
Stress–strain curves of the vulcanizates.

Figure 7 and Table 7 present the mechanical properties of the vulcanizates. In the stress–strain curve, the 300% moduli show the same trend as that of the total crosslink density of the compounds. The moduli of the E-LqIR compounds are smaller than that of the TDAE oil compound because the consumption of sulfur resulted in a lower total crosslink density in the E-LqIR compounds. In the E-LqIR compounds, the crosslink density slightly decreased as the epoxide contents increased, so the 300% moduli were also slightly lowered, but showed a generally similar value.

The wear resistance of the compounds was evaluated through DIN abrasion tests, and the corresponding results are listed in Table 7. The E-06 compound, with high filler–rubber interactions, demonstrated superior wear resistance, although its total crosslink density was lower than that of the TDAE oil compound [12,34]. However, in the case of E-22 and E-34, as the epoxide content increased, the filler–rubber interactions decreased because the E-LqIRs acted as covering agent instead of coupling agents. Therefore, the E-22 and E-34 compounds showed low wear resistances, compared to that of the TDAE oil compound, owing to the lower total crosslink density in the E-LqIR compounds.

### 3.5. Dynamic Viscoelastic Properties

**Figure 8:**
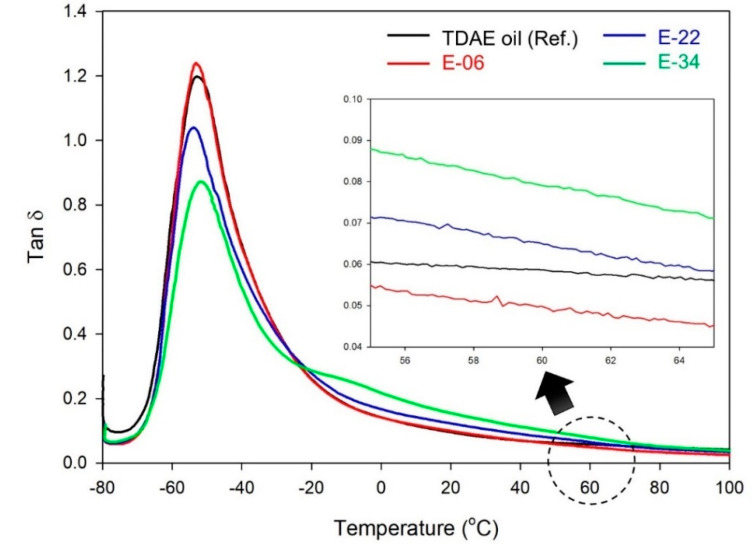
Temperature dependence of tan δ for the vulcanizates.

Figure 8 and Table 8 show the results of dynamic viscoelastic property analysis. The tan δ value at 60 °C is an indicator of the rolling resistance (RR) of a tire. A lower RR value indicates higher fuel efficiency [35]. In addition, the loss modulus (*E*″) at 0 °C is an indicator of the wet grip performance of a tire, and a higher value indicates better wet grip performance [36,37].

Typically, rubber compounds exhibit a high tan δ value near *T_g_* because of the hysteresis of the rubber chains [34]. Moreover, the tan δ value at 60 °C is generally more affected by the agglomeration and deagglomeration of silica networks than by the hysteresis of rubber; as a result, the tan δ value decreases as the dispersion of silica increases [34]. However, the *T_g_* of the E-LqIR increases with increasing epoxide content. Thus, the tan δ peak of the E-LqIR compounds shifts to a lower value and broadens with the increasing epoxide content [38,39]. Accordingly, the value of tan δ at 60 °C increased as the peak of tan δ became broader. On the other hand, the value of *E*″ at 0 °C increased with increasing epoxide content because of the broader rubber hysteresis, indicating a superior wet grip performance. The E-06 compound did not show any significant effect on the *T_g_* because of its low epoxide content; as a result, E-06 compound showed a low value of tan δ at 60 °C compared to that of the TDAE oil compound. These results were obtained because of the superior silica dispersion and higher filler–rubber interactions in the E-06 compound. Thus, compared to the fuel efficiency of the TDAE oil compound, the E-22 and E-34 compounds, owing to the effect of their epoxide contents on the *T_g_*, exhibited low fuel efficiencies, while the E-06 compound exhibited a comparatively higher fuel efficiency.

## 4. Conclusions

In this study, the effects of the epoxide content on the properties of silica-filled NR vulcanizates with E-LqIR as processing aids were confirmed. As the epoxide content of the E-LqIRs increased, the interactions between the epoxide group and the hydroxyl group of silica increased, confirming that the silica dispersion was improved with increasing epoxide content. Increasing the epoxide content also increased the resistance to extraction. Utilizing this effect can reduce the problem of oil migration in tires. The analysis of the vulcanizate structure exhibited that the chemical crosslink density increased with increasing epoxide content, and the E-LqIR acted as a covering agent; however, the filler–rubber interactions decreased because the crosslinking of NR by sulfur was reduced as a result of the smaller number of double bonds in the E-LqIRs. Consequently, the E-06 compound demonstrated the highest filler–rubber interactions and wear resistance.

The addition of E-LqIRs increased the *T_g_*, and the corresponding peaks became broader with the increasing epoxide content. As a result, an improvement in the wet grip performance is expected because of the increased *E*″ at 0 °C for high epoxide contents; however, a low fuel efficiency is expected as well because of the simultaneous increase in tan δ at 60 °C. On the other hand, the effect of the E-LqIRs on the *T_g_* was not significant in the E-06 compounds, owing to the low epoxide content. Furthermore, the higher filler–rubber interactions of the compounds reduced the hysteresis of silica. As a result, the E-06 compound exhibited a lower tan δ at 60 °C compared to that of the TDAE oil compound.

The results of this study showed that a high-epoxide-content E-LqIR could solve the oil migration problem because of the higher interactions of E-LqIRs with silica, and a superior wet grip performance is also expected because of the increased *T_g_*. However, the E-LqIRs that act as covering agents, rather than as coupling agents, decrease the wear resistance. Additionally, the high *T_g_* due to the E-LqIRs leads to low fuel efficiency. Therefore, it was determined that the E-06 compound with a low epoxide content would provide the highest wear resistance and fuel efficiency because it does not affect tan δ at 60 °C and simultaneously exhibits high filler–rubber interactions.

## Figures and Tables

**Table 1:** Formulation of the NR/silica compounds using processing aids (unit: phr).

	TDAE Oil	E-06	E-22	E-34
NR	100	100	100	100
Silica (Ultrasil 7000GR)	55	55	55	55
TESPT ^(1)^	4.4	4.4	4.4	4.4
TDAE oil	10	-	-	-
E-LqIR ^(2)^	-	10	10	10
ZnO	4	4	4	4
Stearic acid	3	3	3	3
6PPD	2	2	2	2
TMQ	1	1	1	1
Sulfur	1.5	1.5	1.5	1.5
CBS	1.5	1.5	1.5	1.5
DPG	1.06	1.06	1.06	1.06
PVI	0.3	0.3	0.3	0.3

^(1^^)^ amount of silane coupling agent was calculated as 8 wt% of the weight of silica. ^(2)^ with different epoxide contents were used.

**Table 2:** Mixing procedure used to prepare the compounds and vulcanizates.

	Time (min:s)	Action
First stage	0:00–1:30	NR mastication (initial temp.: 100 °C)
	1:30–2:40	Add silica 1/2, TESPT 1/2, and oil or E-LqIRs 1/2
	2:40–3:40	Add remaining silica, TESPT, and oil or E-LqIRs
	3:40–5:30	Add ZnO, St/A, 6PPD, and TMQ
	5:30	Ram up
	5:30–7:30	Extra mixing and dump (dump temp.: 150–155 °C)
Second stage	0:00–0:30	Master batch from first stage (initial temp.: 50 °C)
	0:30–2:30	Add curatives and dump (dump temp.: 80–90 °C)

**Table 3:** Characteristics of the LqIR and E-LqIRs.

	LqIR	E-LqIRs		
		E-06	E-22	E-34
*M*_n_ (g/mol)	3639	3761	4200	4401
Polydispersity	1.10	1.09	1.10	1.12
3,4-Addition (mol%)	14.7	14.0	14.8	14.6
1,4-Addition (mol%)	85.3	80.0	63.1	51.0
Epoxide contents (mol%)	0	6.0	22.1	34.4
*T*_g_ (°C)	−77.60	−72.46	−60.28	−47.44
Functionality (epoxide/chain)	0	3.3	13.0	20.6

**Table 4:** Payne effect.

Compounds	TDAE Oil	E-06	E-22	E-34
Δ*G*′ (0.28–40.04%, MPa)	0.65	0.58	0.49	0.48

**Table 5:** Curing characteristics and Mooney viscosities of the compounds.

Compounds	TDAE Oil	E-06	E-22	E-34
Mooney viscosity (ML_1+4_@100 °C)	56.7	53.7	49.8	48.9
Bound rubber contents (%)	32.5	31.3	29.1	28.1
*T*_min_ (N-m)	0.18	0.16	0.13	0.12
*T*_max_ (N-m)	1.52	1.41	1.35	1.31
Δ*T* (N-m)	1.34	1.25	1.22	1.19

**Table 6:** Weight loss and crosslink densities of the vulcanizates (units: 10^−5^ mol/g).

Compound	TDAE Oil	E-06	E-22	E-34
Weight loss after extraction (wt%)	8.48	7.24	6.52	5.89
Weight loss after extraction of 10 phr TDAE oil or E-LqIRs (%)	100	74.5	59.7	46.7
Total crosslink density (chemical crosslink density + filler–rubber interaction)	9.66	9.08	9.01	8.99
Chemical crosslink density	7.26	6.25	6.43	6.54
Filler–rubber interaction	2.40	2.83	2.58	2.45

**Table 7:** Mechanical properties of the vulcanizates.

Compound	TDAE Oil	E-06	E-22	E-34
M_100%_ (kgf/cm^2^)	21.0	19.2	19.4	20.3
M_300%_ (kgf/cm^2^)	77.2	72.0	69.3	68.3
Elongation at break (%)	690	710	680	700
Tensile strength (kgf/cm^2^)	279	275	261	264
DIN abrasion loss (mg)	152.3	149	162.5	168.5

**Table 8:** Viscoelastic properties of the vulcanizates.

Compound	TDAE Oil	E-06	E-22	E-34
Peak of tan *δ*	1.20	1.24	1.04	0.87
*E*″ at 0 °C (MPa)	1.74	1.44	2.11	4.29
tan *δ* at 60 °C	0.059	0.050	0.065	0.079

## Data Availability

Data presented in this study are available on request from the cor-responding author.
